# Benign phyllodes tumor with hemorrhagic cyst in a 14-year-old girl: A case report

**DOI:** 10.1016/j.ijscr.2020.01.037

**Published:** 2020-02-06

**Authors:** Kimiyasu Yoneyama, Motohito Nakagawa, Asuka Hara

**Affiliations:** Department of Breast Surgery, Hiratsuka City Hospital, Hiratsuka, Japan

**Keywords:** CT, computed tomography, Phyllodes tumour, Breast, Paediatric, Benign tumour

## Abstract

**Introduction:**

The incidence of mammary phyllodes tumor is less than 0.3–0.5% of all breast tumors. Only a few pediatric cases have been reported. Here we report a benign phyllodes tumor that occurred in a 14-year-old girl.

**Presentation of case:**

A left breast mass with a bleeding cyst of about 9 cm was resected after the tumor had rapidly increased in size over the course of 2 months. Imaging examinations suggested an intracystic tumor. Histopathological examination, however, revealed a benign phyllodes tumor without cytomorphism or mitotic figures. The tumor was accompanied by partial bleeding, which was considered to be the cause of the rapid increase in size of the hemorrhagic cyst. The patient remains under observation, and local recurrence has not occurred as of 3 years after surgery.

**Conclusion:**

Due to the rarity of the condition in children and adolescents, an individualized, case-based approach is recommended.

## Highlights

• A rare phyllodes tumor in a 14-year-old girl.
• It increased rapidly due to bleeding and was accompanied by cyst formation.
• Needle biopsy is also important for differential diagnosis.

## 1 Introduction

The incidence of mammary phyllodes tumor is less than 0.3–0.5%, making it a relatively rare disease [[1], [2], [3]]. Phyllodes tumor has been found in patients of various ages ranging from 10 to 70 years, but the peak incidence occurs in patients between 40 and 50 years of age. This tumor is rarely observed in adolescents, with only 20 cases reported to date [4]. Juvenile fibroadenoma is a relatively common mammary tumor in young people and grows rapidly to form a large mass. In rare cases, however, such lesions might actually be phyllodes tumors. When needle biopsy fails to provide a definite preoperative diagnosis, excision is usually performed. Surgery for benign lesions of the mammary gland requires care, taking into consideration both curability and functional preservation. Particularly in girls and young women who may wish to breastfeed an infant, invasive treatment should be avoided in consideration of tolerability and mental well-being.

We report a benign phyllodes tumor with a hemorrhagic cyst occurring in a 14-year-old girl with a rapidly growing, bleeding cyst. This work was written in accordance with the SCARE criteria [5].

## 2 Presentation of case

**Fig. 1:**
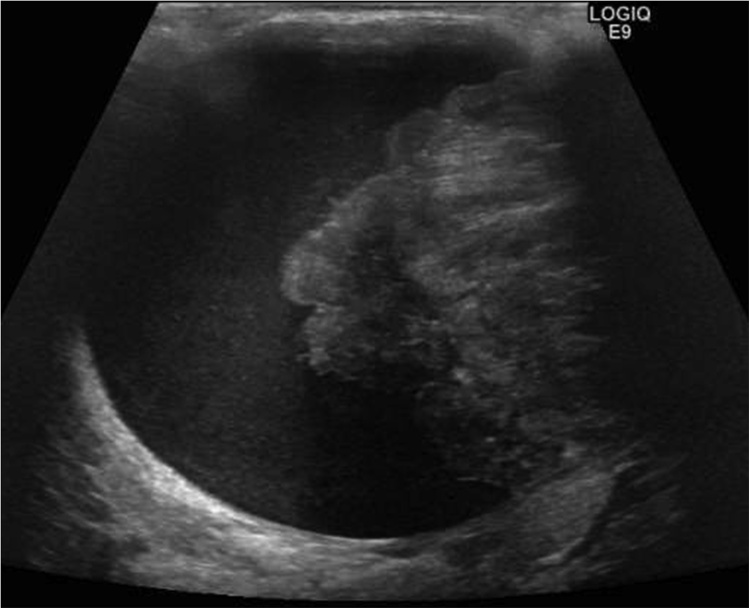
Ultrasonography shows a cystic mass 9 cm in diameter, with a recognizable papillary solid component inside.

**Fig. 2:**
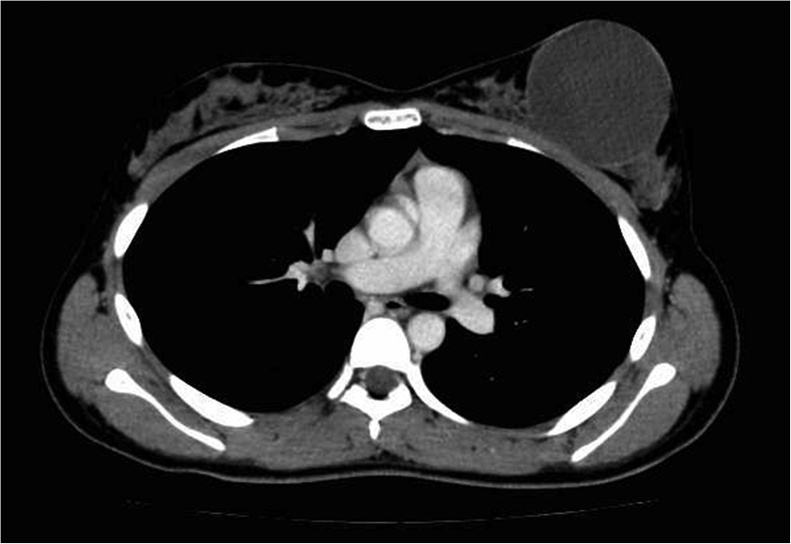
Computed tomography shows a cystic tumor. A papillary stained portion was observed on the wall, but the findings are not as clear as those of ultrasonography.

**Fig. 3:**
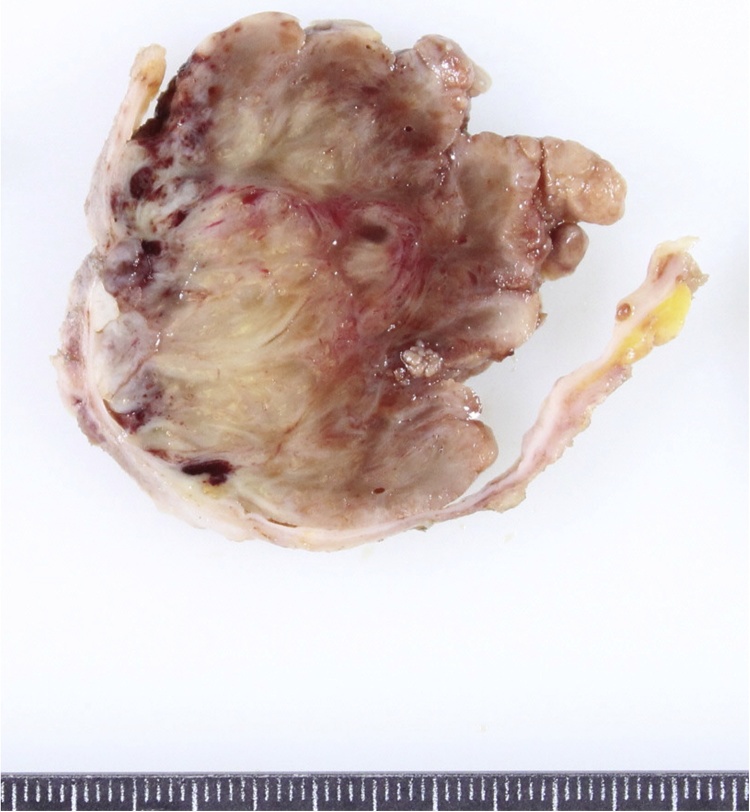
Macroscopic findings of excised specimens. The tumor appears yellowish-white and encapsulated. The cyst wall is broken in cross section.

**Fig. 4:**
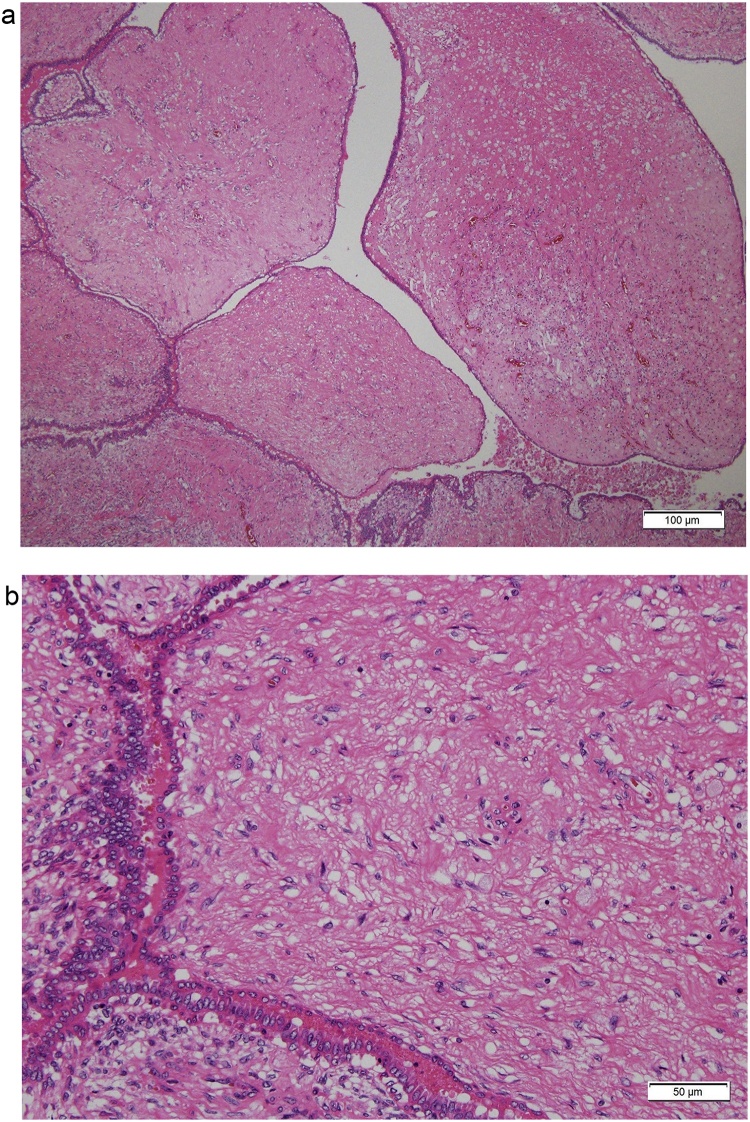
(a) Histopathological findings show a tumor with a leafy structure; hyalinized fibrosis and edema are prevalent in the interstitium. (b) There is no cell heteromorphism and only a few mitotic figures are seen, so the tumor was diagnosed as being a benign phyllodes tumor.

A 14-year-old girl visited our hospital with a left breast mass. She had periodic menstruation and had no family history of breast disease. She had noticed the mass 6 months earlier, and it had dramatically increased in size beginning 2 months earlier. Bloody nipple discharge had also occurred on the day before her visit. A hard elastic mass 9 cm in diameter with good mobility and no tenderness was palpated directly under the left nipple, and the skin directly above was noticeably thinned. The axillary lymph nodes and supraclavicular lymph nodes were not palpable. Ultrasonography revealed a cystic mass 9 cm in diameter, with a recognizable papillary solid component inside (Fig. 1). Computed tomography (CT) revealed a cystic mass including an enhanced papillary component on the wall (Fig. 2). When the cyst was punctured, lavage fluid containing old blood was aspirated. No cellular components were observed, and cytology revealed no malignant findings. Cytology was not performed due to low levels of bloody papillary secretions. A benign cystic tumor with hemorrhage was suspected, and tumor excision was performed. The tumor was removed with care taken not to damage to the cyst wall. The postoperative course was good. Macroscopically, the tumor was yellowish-white and encapsulated. A solid cyst had formed, with the solid part showing proliferation in a papillary state and partial bleeding (Fig. 3). Histopathologically, the tumor had a leafy structure, and marked hyalinized fibrosis and edema in the interstitium. The pathological diagnosis was a benign phyllodes tumor without cytomorphism or mitotic figures (Fig. 4). The tumor was excised without an attached margin, but no additional resection was performed in consideration of the patient’s age and tolerability. Although the patient continues to be followed up in the outpatient clinic, the condition of the breast has remained good and no local recurrence has been noted occurred as of 3 years after surgery.

The patient provided written informed consent prior to publication of this case report and the accompanying images.

## 3 Discussion

Phyllodes tumors account for 0.3% to 0.5% of all breast tumors. Although they can occur in patients over a wide age range, phyllodes tumors most commonly occur in patients in their 40 s, and pediatric onset is rare. They are rarely observed in adolescents, and only 20 cases have been reported; occurrence especially under 14 years of age is rare [6,7].

Phyllodes tumors are often palpated as a hard elastic mobile mass. The clinical features of phyllodes tumors include a rapid increase in size. They share similarities with giant fibroadenoma, which is a more serious disease. In general, it is difficult to differentiate between phyllodes tumor and juvenile (giant) fibroadenoma. Typical phyllodes tumors are circumscribed, oval, and hypoechoic solid masses on ultrasonography [8]. A peripheral cystic component and cleft may be seen more often in phyllodes tumor than in fibroadenoma. In the present case, ultrasonography and CT examination showed that most of the tumor mass occupied the cyst region, so the mass was diagnosed as being a cystic tumor. The cyst was filled with blood, presumed to be due to bleeding from the tumor. MRI may have been useful for discriminating cyst region from solid tumors.

Phyllodes tumor of the breast can be classified as benign, borderline, or malignant based on features such as necrosis, margins (pushing or infiltrative), cellular atypia, stromal overgrowth, and the number of mitoses per high power field [9]. Although 85% of phyllodes tumors in children and adolescents are benign, cases of infiltration, metastasis, or recurrence have been reported, with a mortality rate of about 3% [8].

Pathological examination is essential for a definitive diagnosis. Imaging examinations in the present case resulted in a preoperative diagnosis of intracystic tumor, but the diagnosis was changed to benign phyllodes tumor after examination of the excised specimen.

The present case reaffirms the importance of performing needle biopsy before surgery, for both differential diagnosis and the selection of an appropriate procedure.

Surgical excision is the principal treatment for phyllodes tumor. If the tumor is diagnosed as phyllodes tumor on biopsy, wide surgical excision with a safety margin of 1–2 cm should be performed regardless of histologic subtype [8]. A recent study by Yom et al. concluded that a clear margin of 0.1 mm is equivalent to a margin of 1 cm [10]. The prognosis is generally good. However, recurrence occurs even after complete resection in at least 20% of benign phyllodes tumors [11,12], and the risk of malignancy increases by about 8% with each recurrence [13].

In the present case, since the preoperative diagnosis was intracystic tumor, excision of the mass was performed. The diagnosis was benign phyllodes tumor, and the resection margin was negative. However, even benign phyllodes tumor has a local recurrence rate of about 20%, so careful follow-up is necessary after surgery. Recurrence can develop more than 2 years after surgery, and the present case will require strict follow-up observation in the future. As of 3 years after the surgery, however, no recurrence has been noted and tolerability has been good.

Surgery for benign diseases of the mammary gland requires care taking into consideration both curability and functional preservation. Particularly in girls and young women who may wish to breastfeed an infant in the future, excessive invasive treatment should be avoided in consideration of tolerability and mental well-being, and dissection of the mammary gland and duct should be minimized [14]. Even if the present case had been diagnosed as phyllodes tumor before surgery, the range of ablation should have still been kept to a minimum.

Fibroadenoma is the most commonly observed breast tumor in pediatric patients, but phyllodes tumor should be considered as a differential diagnosis if a rapid increase in tumor size is observed, as in the present case.

## 4 Conclusion

To ensure optimal treatment, careful decision-making regarding the treatment policy, including strict follow-up observation, is necessary. A case-based, individualized approach is recommended, as there is currently no set protocol.

## Sources of funding

Our study has not received any grant of funding.

## Ethical approval

Our institution does not require ethical approval for a case report that are deidentified and collected retrospectively.

## Consent

Written informed consent was obtained from the patient and her mother for publication of this case report and accompanying images.

## Author contributions

Kimiyau Yoneyama contributed to operation and writing the manuscript.

Asuka Hara contributed to operation.

Motohito Nakagawa reviewed the work.

## Declaration of Competing Interest

The authors declare that there is no conflict of interest regarding the publication of this article.
